# Exploiting Pharmacokinetic/Pharmacodynamic Methods for Optimizing and Accelerating Drug Development of Innovative Anti‐Infectives

**DOI:** 10.1002/cmdc.202500743

**Published:** 2026-01-31

**Authors:** Katharina Rox

**Affiliations:** ^1^ Department of Chemical Biology Helmholtz Centre for Infection Research (HZI) Inhoffenstrasse 7 38124 Braunschweig Germany; ^2^ Partner Site Hannover‐Braunschweig German Center for Infection Research (DZIF) Inhoffenstrasse 7 38124 Braunschweig Germany

**Keywords:** anti‐infectives, antivirulence, pathoblocker, pharmacodynamics, pharmacokinetics, pharmacokinetic/pharmacodynamic, proteolysis‐targeting chimeras

## Abstract

Bearing the increase in antimicrobial resistance as well as the emergence of novel viruses with pandemic potential in mind, it is obvious that development pathways need to be accelerated further. At the same time, attrition risks need to be minimized to allow that drugs reach the patient. For successful translation of novel anti‐infectives, several obstacles have to be overcome. This article illustrates recent developments and advances on pharmacokinetic (PK)/pharmacodynamic (PD) methods that can be employed to optimize preclinical development. It specifically emphasizes PK/PD considerations not only for classical antibacterials and antivirals but also for novel approaches, such as proteolysis‐targeting chimers, click‐to‐release systems, or anti‐virulence concepts. Particularly, the latter, nontraditional anti‐infective solutions pose novel challenges for PK/PD, as development pathways are not yet straightforward. Thus, this article also aims to provide ideas on how to tackle challenging PK/PD aspects of nontraditional anti‐infectives, taking advantage and inspiration from traditional development pathways.

## Graphical abstract

This article focuses on the interplay of absorbed, distributed, metabolized, and excreted (ADME) properties; in vivo pharmacokinetics (PK); and pharmacodynamics (PD) supported by in silico PK/PD modeling techniques to improve drug development by acceleration of processes and reducing attrition risks. It provides insights on standard workflows and ways for development of novel treatment approaches such as proteolysis‐targeting chimer.

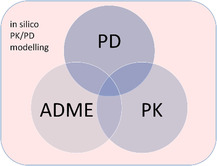

## 1 Introduction

**Scheme 1:**
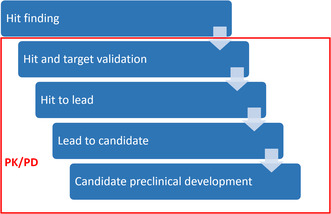
Processes during drug development toward clinical application comprising hit finding, hit and target validation, hit‐to‐lead phase, lead‐to‐candidate phase, and candidate preclinical development (top to bottom) and involvement of PK/PD marked with a red box.

For successful translation of a compound into a drug, pharmacokinetics (PK) and pharmacodynamics (PD) play a crucial role (**Scheme** **1**). They provide an understanding of how a compound is absorbed, distributed, metabolized, and excreted (ADME) and describe the magnitude and type of effects (on specific targets) impacting disease outcome.^[^ ^1^ ^]^ Albeit these disciplines being rather target‐agnostic in general, particular challenges are faced in the context of infectious diseases, such as resistance development, biofilms, persistence, or immune escape.^[^ ^2^, ^3^, ^4^, ^5^, ^6^ ^]^ Depending on the pathogen and the specific target, distinct PK behavior is desired to observe a maximal PD effect. Moreover, the variety of pathogens, for example, viruses, bacteria, fungi, or parasites, renders it difficult to find one general PK/PD correlation fitting all concepts. Thus, individualized and specific solutions are highly desired demanding to not only find novel targets but also innovative targeting approaches.

With decreased research efforts in the field of antimicrobial resistance (AMR) and associated antibacterials as well as antivirals in industry, that is, “big pharma”, these tasks need to be accomplished by small‐ and medium‐sized companies as well as academia.^[^ ^7^ ^]^ This is particularly relevant in light of the estimated further rise in AMR and the associated projected increase in fatality rates.^[^ ^8^, ^9^, ^10^, ^11^, ^12^ ^]^ In 2017, the World Health Organization (WHO) had already introduced a priority pathogen's list to encourage research efforts,^[^ ^13^ ^]^ which had been updated in 2024.^[^ ^14^ ^,^ ^15^ ^]^ A lack of innovation for novel targets had been claimed when the first WHO's priority pathogens’ list was introduced.^[^ ^16^, ^17^, ^18^ ^]^ However, recent examples provide highlights on either novel targets resulting in bacteriostatic or bactericidal effects^[^ ^19^, ^20^, ^21^, ^22^, ^23^, ^24^, ^25^, ^26^, ^27^, ^28^, ^29^, ^30^ ^]^ or on innovative concepts, such as antivirulence,^[^ ^31^, ^32^, ^33^, ^34^, ^35^, ^36^, ^37^, ^38^ ^]^ “Trojan‐horse”,^[^ ^39^, ^40^, ^41^, ^42^, ^43^ ^]^ or click‐to‐release strategies.^[^ ^44^ ^]^ In particular, for the latter nontraditional approaches, that is, antivirulence, Trojan‐horse, or click‐to‐release strategies, it is thought that they constitute useful additions complementing the anti‐infectives repertoire. Specifically, it is assumed that antivirulence approaches disarming bacteria will result in less or no resistance development and render highly resistant pathogens susceptible again.^[^ ^45^ ^,^ ^46^ ^]^ Likewise, Trojan‐horse strategies are employed in particular for Gram‐negatives taking advantage of the siderophore system and bringing antibacterial cargo into the bacterial cell—leading to efficient killing.^[^ ^43^ ^,^ ^47^ ^,^ ^48^ ^]^ Finally, click‐to‐release‐systems are a relatively new concept and aim to provide antibacterial efficacy only at the desired site of action. This enables a precision‐medicine approach with the hope of less resistance development, and in case of antibacterials, harboring adverse side effects, less toxicity concerns.^[^ ^44^ ^,^ ^49^ ^,^ ^50^ ^]^

Likewise, also for antivirals increased research efforts are needed, as recently illustrated by a prioritization list for pathogens with pandemic potential prepared by the WHO.^[^ ^51^, ^52^, ^53^ ^]^ Whereas, the research community in the sense of a pandemic preparedness strategy focuses on respiratory pathogens, also emerging viruses are in the spotlight, as they start to move north and cause considerable disease burden in endemic regions.^[^ ^54^, ^55^, ^56^, ^57^, ^58^ ^]^ The majority of antivirals in the market currently relies on traditional mechanisms, such as inhibition of replication, transcription, entry, or translation, bearing risks for resistance development. By contrast, the main protease in SARS‐CoV‐2 has been proven to be a quite conserved target.^[^ ^59^, ^60^, ^61^, ^62^, ^63^, ^64^, ^65^, ^66^, ^67^, ^68^ ^]^ Apart from traditional direct‐acting antivirals, proteolysis‐targeting chimeras (PROTACs) have led to a paradigm shift in drug discovery recently. As PROTACs degrade their target and act catalytically, sub‐stoichiometric dosing is possible with—depending on the target protein to be degraded—longer lasting PD effects compared to traditional small molecule inhibitors.^[^ ^69^, ^70^, ^71^, ^72^, ^73^, ^74^ ^]^ To date, they have only been explored for a minority of viral targets, but this is expected to increase.^[^ ^75^, ^76^, ^77^, ^78^, ^79^, ^80^, ^81^ ^]^

Apart from target‐based considerations affecting the magnitude of the PD effect, which is crucial to illuminate in particular for PROTACs and click‐to‐release strategies, the diversity of therapeutic approaches also bears challenges from a PK perspective. This is seen for natural products, independent of the mechanism of action, for “Trojan‐horse” strategies, frequently harboring high molecular weight and not following “Lipinski's rule of five”,^[^ ^82^ ^]^ either, as well as for PROTACs. In the following traditional preclinical tracks for small molecules will be illustrated. Moreover, special emphasis will be given on recent developments for PK/PD methods to reduce attrition rates, optimize, and accelerate development. Finally, recent challenges and potential solutions from a PK/PD perspective for PROTACs, click‐to‐release, and antivirulence agents will be discussed.

## 2 Traditional Drug Development Path for “Classical” Anti‐Infectives and Implications for Novel Approaches

Understanding PK/PD relationships is essential for successful translation—independent of “classical”, that is, direct‐acting antibacterials, antivirals, or novel modes of action. This provides the basis to decipher, which PK behavior drives the PD effect or which PK behavior is desirable to achieve maximal PD effect. Finally, this enables to provide recommendations for dosing and dosing interval.^[^ ^83^ ^]^

To enable and to provide a magnitude for these PK/PD relationships, so‐called PK/PD drivers have been established around 20 years ago for the field of direct‐acting antibacterials.^[^ ^84^ ^]^ Essentially, three major possible PK/PD index categories were observed, all relating PK parameters to the in vitro determined minimal inhibitory concentration (MIC) as a measure of efficacy, that is, PD effect: (1) (*f*)*C*_max_/MIC, (2) (*f*)area under the curve (AUC)/MIC, or (3) %(*f*)*T* > MIC. Thereby, all three indices either employ the total or unbound (*f*) concentrations or exposures. The first index, *C*_max_/MIC, describes that efficacy is driven mostly by maximal concentration, *C*_max_, whereas the second index, AUC/MIC, emphasizes that a certain exposure, AUC, is needed for efficacy. The third index, %*T* > MIC, describes the percentage of time for a concentration being above the in vitro determined MIC.^[^ ^84^ ^]^ Furthermore, it has been observed that the same class of antibacterials harbors typically the same PK/PD index. For example, penicillins harbor a %*T* > MIC index, whereas aminoglycosides are exposure‐dependent (AUC/MIC).^[^ ^85^ ^]^ This PK/PD index concept massively facilitates development as PK/PD relationships can be determined for a compound representative of an entire class already at an early stage, whereas the development program can focus on optimizing PK. Results from PK/PD relationship‐determination can then be used for the optimized molecule to provide a proof‐of‐concept and run pivotal trials. It has to be emphasized that the PK/PD index is a universal concept: an index determined in an in vivo or in vitro infection model will be the same as in the clinic, the magnitude of the index, however, will differ.^[^ ^84^ ^]^ For successful translation of PK/PD indices from animal to human, in vitro and in vivo data are incorporated into PK/PD models to select the dose and dosing regimen for treating different types of infection.^[^ ^86^ ^,^ ^87^ ^]^ Apart from these three major indices, recent investigations aimed to elucidate additional PK/PD relationships, reflected by novel PK/PD drivers.^[^ ^88^ ^]^ Additionally, the PK/PD driver concept has been extended using semi‐mechanistic modeling for dose estimation to suppress emergence of resistance.^[^ ^89^ ^]^ Furthermore, recently, semi‐mechanistic PK/PD models have been employed instead of experimentally determined PK/PD indices to improve dose selection in distinct patient populations, harboring challenges for PK, and subsequently, of efficacy because of physiological alterations due to either age, obesity, or conditions underlying the infectious disease.^[^ ^90^, ^91^, ^92^, ^93^ ^]^

Surprisingly, PK/PD relationships are mainly established in the presented, systematic manner of PK/PD indices for antibacterial drug development (direct‐acting antibacterials). Considering other areas for anti‐infective drug development, such as antivirals or antiparasitic drugs, these “clear” relationships are not investigated systematically. For antivirals, typically the IC_50_, EC_50_ or EC_90_ serve as correlate instead of the MIC (in case of antibacterials). However, the PK/PD index concept is in principle also conceivable for antivirals.^[^ ^94^ ^,^ ^95^ ^]^ Unfortunately, because development of novel antivirals is rather based on achieving high plasma or tissue concentrations in relation to the in vitro determined PD efficacy marker, PK/PD indices for antivirals are often not studied in‐depth. Consequently, for antivirals, the translational connection from preclinical to human is not established in the same way as for direct‐acting antibacterials. It also implies an increased burden for the development of antivirals: because the PK/PD relationship is unknown for distinct classes, this demands a very good safety/toxicity profile. Moreover, antiviral drug development comes with an additional level of complexity: either rodent models are not available or not completely reflecting human pathology rendering translation and model selection even more demanding.^[^ ^96^, ^97^, ^98^ ^]^

**Scheme 2:**
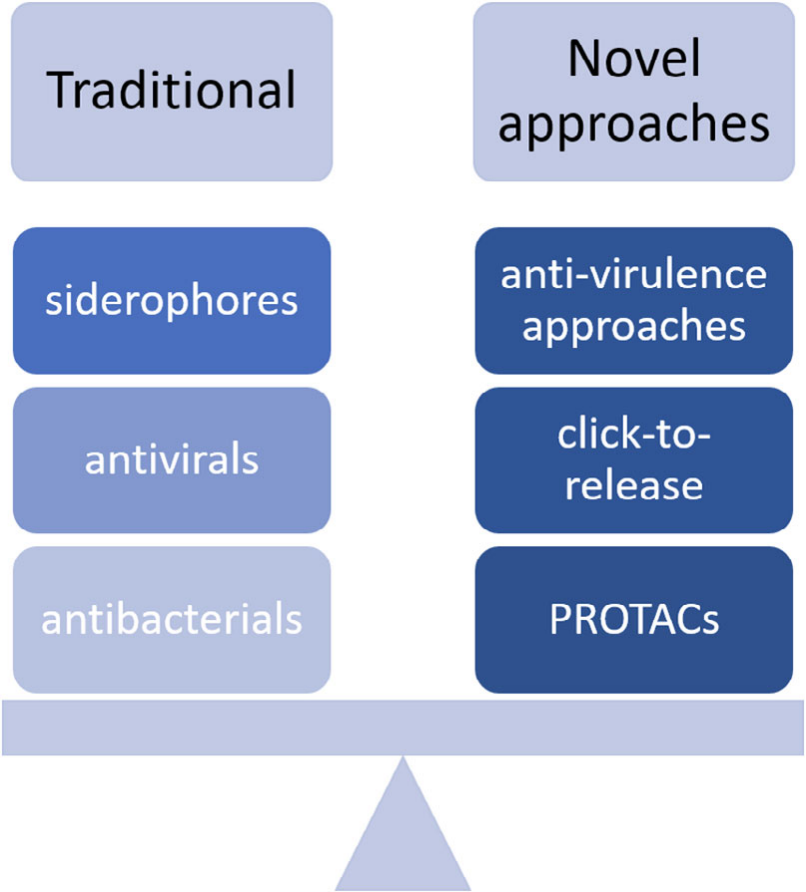
Development pathways for different treatment approaches grouped by mechanism of action. The ‘traditional’ development pathway can be followed for direct‐acting antibacterials and antivirals as well as for siderophores and Trojan‐horse strategy approaches with respect to PK/PD drivers. For PROTACs, click‐to‐release‐systems, and antivirulence approaches, novel PK/PD relationships need to be elucidated.

Potential PK/PD relationships for novel treatment approaches, such as “Trojan‐horse” strategies, represented by siderophores, PROTACs, or antivirulence agents, have to be closely examined (**Scheme** **2**). For cefiderocol, as a siderophore representing the “Trojan‐horse” strategy, the same PK/PD index as observed for cephalosporins has been described.^[^ ^99^ ^]^ Thus, challenges might be rather associated with PK behavior because of *inter alia* the molecular weight.^[^ ^82^ ^]^ It can only be hypothesized that this classical PK/PD concept based on the antibacterial linked to the siderophore is still valid, as sufficient examples to prove that connection are still missing. However, the observation that cefiderocol's efficacy is described by a PK/PD index seen for cephalosporins is encouraging for providing a defined PK/PD path forward. When considering PROTACs, PK/PD relations still need to be clarified as they depend on the extent of degradation. This will be discussed in more depth in a dedicated section herein (*vide infra*). Finally, antivirulence approaches have to be carefully examined as they might target factors on surfaces or secreted proteins. First, a suitable biomarker or measure of PD effect needs to be elucidated to enable in‐depth PK/PD relation investigations.

Independent of the therapeutic approach and chemical structure, a thorough ADME and PK characterization combined with high‐level information from in vitro and in vivo experimentation is required to, finally, add mechanistic knowledge of biological processes. This degree of information will ultimately enable to reduce attrition risks and improve the information for safety assessment.^[^ ^100^ ^]^

### 2.1 Assessment of ADME Properties Providing a Guide for Selection

**Scheme 3:**
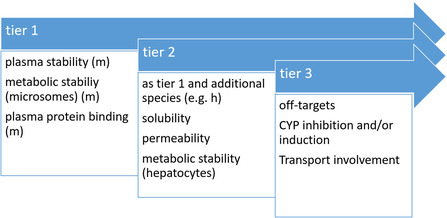
A tiered approach for assessment of the majority of ADME parameters is displayed, which can vary project dependently. In essence, ADME profiling starts with a limited set comprising plasma stability, metabolic stability, and plasma protein binding, followed by solubility, permeability, and metabolic stability assessment in hepatocytes during tier 2. Finally, for candidate selection off‐targets, and if suspected, CYP inhibition and/or induction or transport involvement is assessed.

For translational success, also PK/PD and target‐based considerations and evaluations have to be closely aligned. The first step for PK assessments in vitro, are so‐called ADME assays laying the foundation and providing a stratification matrix for further development. Thus, initial ADME properties are already assessed at the hit‐stage and are investigated in more detail from lead to candidate compound (**Scheme** **3**).^[^ ^100^ ^]^

The primary aim of ADME assays is to find a compound with “drug‐like” properties and low safety concerns.^[^ ^1^ ^,^ ^101^ ^]^ Lipinski's “rule of five” gives guidance for “drug‐likeliness” of small molecules, that is, the molecular weight cutoff, the number of hydrogen bond donors as well as acceptors, and the logP value as a measure of lipophilicity for a molecule,^[^ ^82^ ^]^ whereas Veber's rule adds specifically required properties for achieving peroral (PO) bioavailability.^[^ ^102^ ^]^ However, several therapeutic approaches violate these rules, but are still effective therapeutics. Among these are natural products harboring high lipophilicity as well as high molecular weight. Likewise, this applies to siderophores and PROTACs. This illustrates that the “rule of five”^[^ ^82^ ^]^ provides a first framework. Nevertheless, apart from these rules, successful therapeutics can still be developed. A prerequisite is a thorough investigation of ADME properties.

In general, the in vitro ADME set comprises assessment of plasma protein binding, plasma stability, metabolic stability, cytotoxicity, lipophilicity, blood‐to‐plasma distribution coefficient, solubility, and permeability through membranes.^[^ ^1^ ^]^ It has to be emphasized that not the entire method set is used during early stages of medicinal chemistry optimization programs. Thus, rather a tiered approach is followed: during hit‐to‐lead stage cytotoxicity, plasma protein binding, plasma stability, and metabolic stability might only be assessed in one species, whereas during lead‐to‐candidate stage, also metabolite identification (MetID) studies, solubility and lipophilicity are determined. For selection of a development candidate additional parameters, such as CYP inhibition/induction studies, mutagenicity testing, safety panels (comprising hERG channel inhibitory properties) as well as blood‐to‐plasma distribution coefficient are determined (Scheme 3).^[^ ^1^ ^,^ ^103^ ^,^ ^104^ ^]^

Metabolic assessments are typically performed using microsomes, S9‐fractions, or hepatocytes. They serve to understand the different phases (phase I or phase II) of metabolism of a compound during early stages and allow optimization to confer stability or to exclude harmful metabolites associated with safety concerns, or to deprioritize a series. When MetID studies are employed, they are performed in several species, at least in one preclinical and human species enabling to conduct initial in silico in vitro–in vivo extrapolations. Moreover, MetID studies enable to predict drug–drug‐interactions based on CYP‐enzymes or transporter‐mediated interactions. This aids to conduct a risk assessment for compound selection already at an early stage.^[^ ^105^, ^106^, ^107^ ^]^

Whereas the development goals for metabolic stability are clear, determination of plasma protein binding still remains controversial, although needed to estimate the effect based on the unbound fraction. One reason associated with the difficulty of accurate plasma protein binding assessment is that plasma protein binding of small molecules might change in the context of infections or specific patient characteristics.^[^ ^108^ ^]^ Several methods, such as rapid equilibrium dialysis or ultracentrifugation, are used to measure plasma protein binding in vitro and in vivo.^[^ ^109^ ^]^ However, the exact value is highly dependent on the method chosen. More recent methods, for example, using saturation or dilution, seem to enable to determine the fraction unbound, even for highly protein‐bound drugs.^[^ ^108^ ^]^ Specifically, accurate determination of plasma protein binding levels is crucial for natural products,^[^ ^110^ ^,^ ^111^ ^]^ but also for PROTACs harboring additional ADME/PK challenges, because of *inter alia* low solubility, high binding to microsomes, different elimination pathways, and chameleonic properties.^[^ ^112^, ^113^, ^114^ ^]^

Lipophilicity serves as a first estimation for distribution within the body, but also for membrane permeability and is assessed as logD or logP. The additional measurement of solubility, either via thermodynamic or kinetic measurements, together with permeability determination using established assay systems, that is, PAMPA, CaCo‐2 cells, or MDCK/MDCK‐MDR1 cells, enables to group compounds into the biopharmaceutics classification system (BCS).^[^ ^115^ ^,^ ^116^ ^]^ Chemical strategies, such as prodrug approaches or salt formation, can aid to increase solubility and permeability. However, classification of compounds into BCS II or IV requires focusing on formulation development early in a program if chemical strategies do not provide success.^[^ ^100^ ^]^ The BCS has been established mainly for small molecules, but is also valid for natural products, antivirulence approaches, and siderophores. For PROTACs, it remains challenging to accurately assess solubility and permeability in in vitro assays to enable to classify them correctly. This might require to adapt assay systems for solubility as well as permeability measurements.^[^ ^112^ ^,^ ^114^ ^]^ Finally, ADME property assessment provides a good basis for weighing which compounds to proceed further. Again, as assay systems need to be adapted for PROTACs and even if they are adapted, still might not reflect effects observed in in vivo systems later, strict employment of ADME assay go/no‐go criteria is not recommended in case of PROTACs because of not so stringent in vitro–in vivo correlations for that particular case.^[^ ^114^ ^]^

### 2.2 Understanding PK Behavior as Foundation for Subsequent Efficacy Studies

The assessment of ADME properties serves in the majority of cases (except PROTACs, vide supra) as a good stratification matrix for compounds for in vivo PK assessment. Still, to date, ADME assessments do not enable to accurately predict for every compound maximal concentrations, exposure, half‐life, volume of distribution, or clearance. Thus, in vivo assessment is still needed.^[^ ^117^ ^]^ Typically, PK studies are conducted in mice as first preclinical species, as the mouse is considered to be the lowest phylogenetic organism with sufficient similarity to humans. In this context, it is helpful to correlate in vitro metabolic clearance data with in vivo PK data to reveal additional mechanisms involved, not detected in vitro, such as unexpected metabolism or transporter‐mediated clearance, demanding additional investigation.^[^ ^107^ ^,^ ^116^ ^,^ ^118^ ^]^ To address these observed effects, Varma and colleagues have proposed the extended clearance classification scheme, based on ionization state and molecular weight of a compound as well as on its membrane permeability to estimate hepatic or renal transporter involvement in clearance.^[^ ^119^ ^]^ However, this scheme has not yet been validated and used to explain effects observed for PROTACs, specifically. For other small molecules, it suggests that enhanced clearance might be rooted in active tubular secretion. Likewise, reduced clearance could be attributed to reabsorption or enterohepatic circulation. Additionally, assessment of levels in feces and urine might aid to estimate and gain more confidence in possible excretion pathways and understand underlying mechanisms.^[^ ^119^, ^120^, ^121^ ^]^

Furthermore, when tissue distribution is assessed during PK studies, a compound can be tracked apart from typical plasma‐concentration‐time profiles and help to decide which indications for development are conceivable. Whereas small molecules typically distribute equally (apart from transport‐mediated or other mechanism‐based uptake) between plasma and tissue, it has been observed for a PROTAC recently, that it preferentially distributed to lung or liver tissue rendering assessment of only plasma‐concentration‐time profiles noninformative.^[^ ^122^ ^]^ However, further studies are needed to understand if this is a principle more often seen in PROTACs or if it was attributed to the specific design. Typically, distinct designs for PK studies are employed, starting from intravenous PK studies, conferring 100% bioavailability, and dependent on the efficacy model and the expected bioavailability, continued with the subcutaneous (SC), intraperitoneal (IP), PO, or local administrations, for example, via inhalation, intranasally, intratracheally, or oropharyngeally. In particular, PO PK studies enable to understand if in vitro ADME assays with respect to permeability align with the observed bioavailability. At earlier stages of drug development, this gives information if distinct chemical approaches, for example, structural modifications to enhance solubility and permeability, or formulation development early in a program is deemed necessary, or ultimately, if none of the strategies is successful, a development program needs to be stopped.^[^ ^123^, ^124^, ^125^ ^]^ Even if a compound does not harbor sufficient PO bioavailability, the exploration of the SC or IP routes of administration and/or a combination to reach sufficient concentrations in target compartments in relation to the PK/PD properties can provide useful information to achieve a proof‐of‐concept or proof‐of‐target engagement warranting further preclinical development. In all cases, microsampling methods should be deployed to reduce the total number of animals per PK study because only minimal blood volume is needed attributed to advanced bioanalytical methods.^[^ ^126^, ^127^, ^128^, ^129^ ^]^ Finally, dose escalation PK studies are employed in case nonlinear PK is suspected based on in vitro and first in vivo data results. Nonlinear PK warrants further mechanistic investigation to understand underlying processes. Ceiling effects might be attributed to enzyme or transporter saturation and their impact needs to be estimated for human PK before further preclinical development.^[^ ^105^ ^,^ ^120^ ^]^

When measuring tissue concentrations, it must be mentioned that only homogenized tissue is used for concentration determination requiring tailored bioanalytical methods.^[^ ^130^ ^,^ ^131^ ^]^ Additionally, as homogenized tissue is employed, no information on the exact compartment where a compound was confined is obtained.^[^ ^132^ ^]^ Thus, data on tissue concentrations needs to be valued as informative providing a first estimation whether compound concentrations in total tissue follow plasma kinetics, whether preferential distribution or accumulation, resulting in potential toxic or adverse side effects, is observed. An additional method to get a detailed understanding of tissue distribution is microdialysis, more frequently employed during human studies and currently not feasible during initial preclinical PK studies.^[^ ^133^, ^134^, ^135^ ^]^ By contrast, epithelial lining fluid (ELF) concentrations, obtained via bronchoalveolar lavage, provide specific information about the localization and concentrations of a compound and enable guiding compound optimization to enhance favorable properties. Specifically for compounds targeting the lung, this information is valuable.^[^ ^33^ ^,^ ^90^ ^,^ ^136^, ^137^, ^138^ ^]^ For these specific questions, focused PK studies, providing not only terminal but also different time‐point concentrations enabling to calculate PK properties not only in plasma but also in target compartments, are crucial.

Finally, PK studies can be considered as a critical decision point for moving forward to efficacy, that is, PD, studies, or for further optimization cycles. Thereby, initially, the best suited route for optimal PK/PD relations needs to be found, whereas during lead‐to‐candidate phase and for candidate selection, in‐depth PK profiling is performed to understand tissue concentrations, if necessary, as well as extent of linearity of PK at higher doses.^[^ ^100^ ^]^

### 2.3 Infection Models for Proof‐of‐Concept Studies as Well as for Proof‐of‐Target Engagement to Ultimately Understand PK/PD Relationships

In the recent years, distinct infection models have been established with the purpose to reflect processes observed in the clinics. However, currently, still no model is available to accurately reflect all aspects. A plethora of in vitro models as alternatives to in vivo models have been used to mimic different aspects of infections in humans and to provide missing information for successful translation. In that way, in precision‐cut‐lung slices (PCLS)^[^ ^139^, ^140^, ^141^, ^142^ ^]^ or air‐liquid‐interface (ALI) infection models,^[^ ^143^ ^,^ ^144^ ^]^ the physiological environment is mirrored. However, these models still lack the perspective of dynamically changing compound concentrations, such as determined in PK studies, and thus, these models can only investigate effects based on static concentrations. A model reflecting these dynamic concentrations and also enabling to study not only bacterial kill over time but also serving to investigate dosing regimens necessary to suppress resistance development, is the hollow fiber infection model (HFIM).^[^ ^145^, ^146^, ^147^ ^]^ It consists of a central compartment connected to a hollow fiber cartridge. Concentrations are varied in the central compartment, impacting concentrations in the cartridge. This enables to mimic PK behavior and study PK/PD effects. Because of the cartridge consisting of several fibers, pathogens^[^ ^148^ ^]^ or cells infected with, for example, viruses^[^ ^95^ ^]^ directly inoculated into the fiber can be cultivated and PD readouts can be assessed. Also, HFIM, like other in vitro models, such as PCLS or ALI cultures, has the drawback—even if the possibility exists to connect several cartridges to reflect different compartments—that interorgan connections are missing and that influence on excretion, metabolism, and distinct distribution kinetics cannot be studied. Hence, in vivo animal models, predictive for human infections are one of several puzzle pieces to understand efficacy on the way to successful translation.^[^ ^149^ ^,^ ^150^ ^]^

**Figure 1:**
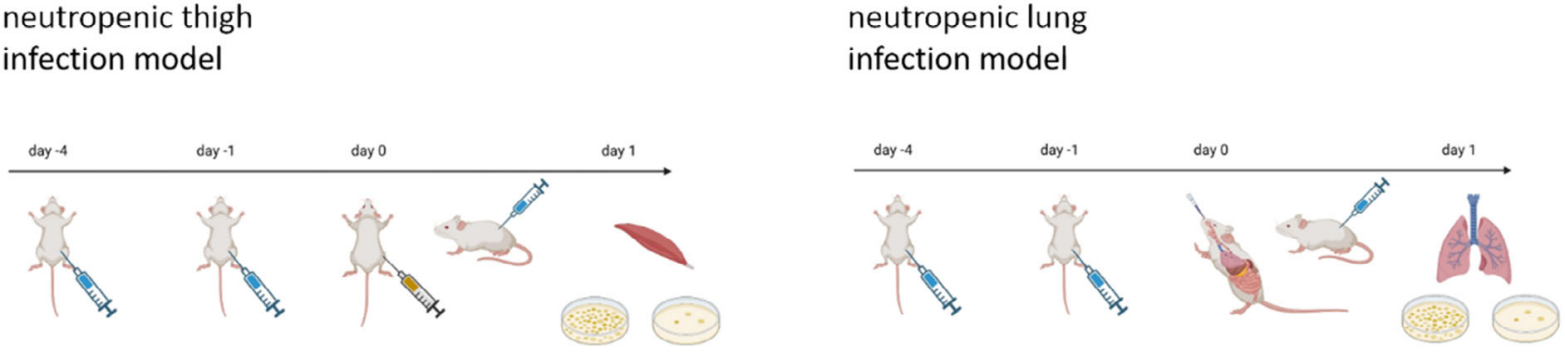
The protocol for the neutropenic thigh (left) and lung (right) infection model is displayed. On day 4 and 1, respectively, animals are rendered neutropenic upon treatment with cyclophosphamide. On day 0, infection is established with either infection into the lateral thigh or into the lung. Treatment is initiated accordingly. On day 1, bacterial burden is either determined in thigh (left) or lung (right). Created in BioRender. Rox, K. (2025) https://BioRender.com/n95c431 and https://BioRender.com/l99o185.

Considering antibacterials, two main “workhorse” models, that is, the neutropenic thigh and the neutropenic lung infection model, have evolved as they provide good predictability for further preclinical and subsequent clinical development (**Figure** **1**).^[^ ^149^, ^150^, ^151^, ^152^, ^153^, ^154^ ^]^ Both models share a similar regimen for rendering animals neutropenic to enable to induce infection for bacterial pathogens and enabling to grow them over time. This allows studying different dosing regimens and routes of administration to deduce PK/PD relationships. An indication for sufficient bacterial growth over time represents the vehicle control group that should demonstrate a significant growth after 24–26 h over the “inoculum control group” as a baseline control group. The third control group is a positive control demonstrating bacterial burden reduction back to baseline (“stasis”) or even below (“cidality”). Compared to in vitro infection models, these models allow to study the direct action of a compound on a bacterial pathogen under the conditions of a changing concentration‐time profile in a physiological environment. Bacteriostatic or bactericidal activity is assessed in relation to the inoculum control group.^[^ ^100^ ^]^ If PK profiles were observed as planned and no PD effect is seen for a compound, then it might need further optimization cycles or a program could be halted. In the opposite case, further assessments are deemed necessary to narrow down potential indications and evaluate the development track. Typically, dose fractionation studies enable to reveal the PK/PD index in vivo and confirm in vitro results.^[^ ^155^, ^156^, ^157^ ^]^ Next, also models more closely mimicking the clinical application, such as soft‐tissue, acute or chronic lung, sepsis, or urinary tract infection models, might be explored depending on the PK behavior observed.^[^ ^29^ ^,^ ^158^, ^159^, ^160^ ^]^

However, the two “workhorse” models lack one important aspect: the involvement of the immune system. Thus, they are not ultimately suited to assess efficacy of novel therapeutic approaches, such as antivirulence agents.^[^ ^161^ ^]^ In that respect, rather immunocompetent models, like acute lung infection models, soft tissue infection models, urinary tract infection models, or chronic lung infection models, might be conceivable. However, these models are complex. First, it is necessary to prove target engagement. Therefore, well‐characterized models are needed, also to enable to demonstrate efficacy or superiority above standard‐of‐care, either in combination or alone. Another aspect associated with the mechanism of action of antivirulence approaches is that bacterial burden as the only readout might not be observed, and thus, not suffice. Hence, distinct readouts are needed calling for antivirulence specific biomarkers. These could be inflammatory markers or specific pathways addressed by the antivirulence agent. For a few antivirulence targets, potential biomarkers have been at least studied in animal models. For example, for the Pqs quorum sensing (QS) system, in *Pseudomonas aeruginosa*, the quinolones 2‐heptyl‐4(1*H*)‐quinolone (HHQ), 2‐heptyl‐4‐hydroxyquinoline N‐oxide (HQNO), and 2‐heptyl‐3‐hydroxy‐4(1*H*)‐quinolone (PQS) as pathway‐specific markers are known. Hence, agents targeting the Pqs QS system should impact HHQ, HQNO, and PQS. One suitable technique is high performance liquid chromatography with tandem mass spectrometry (HPLC‐MS/MS). It has been shown previously that at least in mouse lung, these signal molecules were found and correlated with bacterial burden providing a promising approach for further target engagement studies.^[^ ^162^ ^]^ Another prominent example for antivirulence approaches is targeting the toxin of *Staphylococcus aureus*, targeted by an antibody approach. The inflammatory marker IL‐6 was used to prove the effect and served as a surrogate.^[^ ^163^, ^164^, ^165^, ^166^ ^]^ In a recent study, also a small molecule proved effective in targeting α‐hemolysin. Again, IL‐6 levels were substantially reduced. Additionally, also bacterial burden reduction in the animal model was observed which could be attributed to a more balanced immune response upon treatment.^[^ ^35^ ^]^ Antivirulence agents frequently target protein expression or the protein itself^[^ ^167^, ^168^, ^169^ ^]^ or hamper its dissemination, as observed for a protease secreted by *Pseudomonas aeruginosa*, LasB.^[^ ^170^ ^]^ Hence, beside enzyme‐linked immunosorbend assay (ELISA) assays, also MS/MS techniques for direct protein detection and quantification are conceivable. Matrix‐assistet laser desorption ionization‐time of flight mass spectrometry (MALDI‐TOF‐MS) is typically employed for detection of proteins‐of‐interest by mapping against known spectra of the respective bacterium. Hereby, in clinical practice, bacterial lysates, blood culture, urine, or cerebrospinal fluid are sampled. Also, native mass spectrometry (MS) is used to gain insights into protein–ligand interaction using purified proteins.^[^ ^171^ ^,^ ^172^ ^]^

By contrast, for antivirals, infection models differ quite substantially, attributed to the fact that there is a lack of a common antiviral infection model for all different virus families. Thus, it remains tedious to develop a model that does not only serve as a surrogate, but is also predictive for human infection. For flaviviruses, infection models in IFNAR^‐/‐^ mice as well as in AG129 mice exist, taking advantage of a hampered immune response by knockout of interferon receptors to enable an infection.^[^ ^173^, ^174^, ^175^, ^176^ ^]^ However, these models also come with a significant disadvantage as no viral clearance is observed. Thus, they cannot entirely mimic human infection.^[^ ^176^ ^]^ Therefore, RNA copies of the virus in organs most affected, for example, spleen and liver, are assessed using quantitative polymerase chain reaction (qPCR).^[^ ^177^ ^]^ In case of SARS‐CoV‐2, it was taken advantage of different hosts, such as hamsters, nonhuman primates, and mice. For mice, several mouse strains were employed to reflect the distinct aspects observed in the clinic, such as SCID‐mice, K18‐hACE2, or hACE2‐KI mice.^[^ ^178^, ^179^, ^180^ ^]^ Likewise, for herpesviruses, also distinct models are used. In case of human cytomegalovirus infection, murine cytomegalovirus is used as a surrogate and infection processes are studied. This comes with the limitation that not all aspects of clinical symptoms and inflammatory processes are mirrored. A potential way to enable to provide a better link is to use immunodeficient mouse strains implanted with human cells to enable development of a human‐like immune system. However, refinements and optimizations of these models are still required as host–pathogen interactions are not yet entirely reflected.^[^ ^181^, ^182^, ^183^ ^]^ Nevertheless, for some virus families, adequate and predictive models are still not available, so that regulatory authorities are also open for basing assessments on other predictive models, such as in vitro models or surrogate in vivo models.^[^ ^184^ ^]^ This shall ultimately enable innovation as well as drugs reaching the patient in the end. However, as presented for the antivirals, choosing the right, predictive model remains a challenge—and this is of importance not only for direct‐acting antibacterials or antivirals, but also novel approaches, such as click‐to‐release or PROTACs or antivirulence compounds. Thus, a risk mitigation strategy might consider complementary models, but also taking into account in silico techniques.

### 2.4 Taking Advantage of In Silico Modeling Techniques

It remains an endeavor with unknown success when starting preclinical toward clinical development, also because finding the right infection model providing predictability and enabling proof‐of‐target engagement is cumbersome, particularly for antivirulence approaches, but also for antivirals. In that respect, in silico models are thought to provide essential mosaic parts.

Traditionally, with respect to modeling PK behavior two different approaches exist: compartmental models also employed for population approaches, or more complex whole‐body physiologically based PK (PBPK) models.^[^ ^185^, ^186^, ^187^ ^]^ Compartmental models lack the physiological dimension and are suited to describe PK processes in a simple, fit‐for‐purpose perceived way. They might be perceived as “simpler” compared to PBPK models, but can also increase in complexity when adding different subcompartments. They are frequently used when a compound's PK follows linearity. However, in case of saturation or nonlinear PK, this has to be accounted for. Additionally, compartmental models lack the physiological dimension, which is employed in PBPK models. PBPK models are designed as such every organ is represented with its subcompartimentalization. Additionally, organs are linked via the (virtual) blood flow. This enables to adjust organ and other specific parameters to distinct species and enable cross‐species evaluation of PK properties, as literally “just” physiology and physiological processes need to be adjusted. Thus, PBPK modeling can provide an advantage compared to allometric scaling based on body weight or body surface area, particularly in case of transporter‐ or CYP‐mediated influence on metabolism, distribution, and excretion across species.^[^ ^187^, ^188^, ^189^, ^190^, ^191^, ^192^ ^]^ Because physiological processes, such as renal or hepatic clearance or expression of metabolic enzymes are adjustable as well as plasma protein binding and body water content, disease states or different age‐ranges and the consequences for PK profiles can be explored. This is a valuable option when considering transition from phase I clinical testing in healthy adults and transitioning to later clinical development when patients are enrolled. Thus, PBPK models offer a wide range of possibilities and are well‐suited for translational purposes.^[^ ^193^, ^194^, ^195^ ^]^ Equally, as population PK (popPK) models, compartmental models can be adjusted to explore consequences of, for example, different disease states.^[^ ^196^, ^197^, ^198^ ^]^ Finally, it has to be weighed which level of complexity of a model is required and which final application is envisaged to choose the PK modeling approach.

**Scheme 4:**
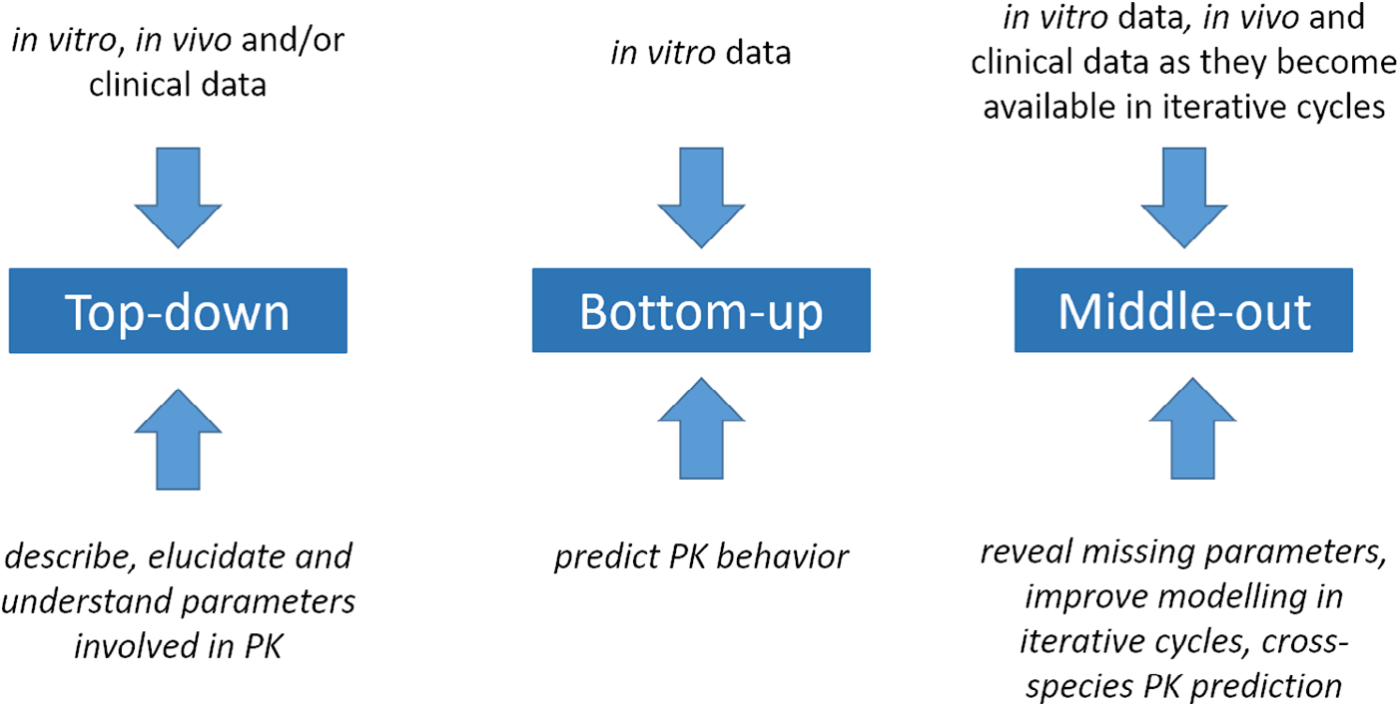
The different PK modeling concepts are displayed. The necessary data input is mentioned on top, whereas the purpose is mentioned at the bottom.

Generally, regarding (PB)PK models, there are three different ways on building models requiring a different degree of available data and serving distinct purposes: (1) “top‐down”, (2) “bottom‐up”, or (3) “middle‐out” approach (**Scheme** **4**).^[^ ^199^, ^200^, ^201^, ^202^ ^]^ The “top‐down” approach aims to construct a (minimal) PK model and needs clinical or other (preclinical) in vivo data as input. By fitting simulated curves to observed data, parameters are adjusted and might provide insights into processes. However, not necessarily deep mechanistic insights are gained, rather this provides hints on potential PK mechanisms needed to be investigated further. In contrast to that the “bottum‐up” approach starts with mainly in vitro data and predicts in vivo outcome. Aspects included in in vitro assay assessment, determine data available for this bottom‐up approach. It can be easily guessed that only a tier‐1 limited ADME setup, comprising only metabolic stability and plasma protein binding, might lead to inaccurate predictions. Hence, ADME assays with a high degree of information illuminating distinct aspects seen in in vivo studies later are required as data input to provide predictions that are more realistic and close to observed data. As this requires an in‐depth mechanistic understanding, it is more common to be performed in the framework of PBPK models. The “middle‐out” approach takes advantage of both aforementioned concepts. It employs a limited number of in vitro data to build initial simulations, frequently in PBPK models. When in vivo data become available, simulations are optimized; potentially elucidating parameters involved that need to be investigated to gather a mechanistic understanding. In iterative cycles, these models are subsequently optimized and extended with more data becoming available. This renders predictions more accurate. Ideally, the models are finally validated with another dataset to evaluate their predictive performance for upcoming studies.^[^ ^200^ ^]^ There are several advantages to incorporate solid and validated (PB)PK models directly in the beginning of preclinical development: (1) PK profiles after administration of different dosing regimens can be predicted and allow estimation of doses necessary for preclinical and clinical studies. Prediction can then serve to reduce experimentation and therefore accelerate the process by mainly running pivotal studies. (2) Mechanistic PBPK models estimate concentrations in tissues and their subcompartments, not assessed during PK studies,^[^ ^132^ ^]^ but important for later PD considerations. Hence, potential toxic, adverse, or side effects (based on in vitro data on off‐target profiling or mechanistic insights, e.g. when accumulation is predicted due to transporter involvement) are estimated prior to a study. (3) Incorporating modeling early in development provides solid models, which can then enable to propose dose adjustments, if necessary, for special populations, such as geriatric, pediatric, obese, or renal‐/liver impaired patients.^[^ ^203^ ^]^ Moreover, several software applications are available to date so that PBPK modeling has been democratized.^[^ ^204^ ^]^ These advantages explain the widespread use of modeling, not only in industry but also in academia to improve compound selection and reduce attrition rate, and finally, costs. However, PK modeling also comes with a challenge and chance that increased communication of distinct disciplines is necessary to build models and to enable to change ancient drug discovery and development workflows.

Apart from dose and dosing regimen prediction, the application of (PB)PK modeling can be further extended by linking it to PD. These PK/PD in silico models shall enable effect prediction and are powerful tools for drug development. In the field of antibacterials, this has been frequently employed by PK/PD modeling.^[^ ^205^, ^206^, ^207^, ^208^ ^]^ In brief detail, plasma concentrations, either preclinical or clinical, were modeled using compartmental PK models and linked to a PD model of bacterial growth. These PD models have also been extended to account for immune components or resistance development under treatment. Likewise, also PBPK models have been linked to PD to investigate the potential of otherwise systemically administered antibacterials for inhalative administration.^[^ ^209^ ^]^ For antivirals, PD models rely on viral kinetic compartmental modeling. In brief, this provides estimates on infected and uninfected cells as well as on components interfering with viral replication, aside from antiviral treatment. These models have been initially developed to describe HIV kinetics, but have been extended for other viruses, such as HCV or SARS‐CoV‐2, recently.^[^ ^210^, ^211^, ^212^, ^213^, ^214^, ^215^ ^]^ To estimate viral replication kinetics for viral PD model construction, an in‐depth understanding is necessary derived from biological experiments. Thus, viral kinetics and impact of immune components are estimated using simulation based on in vitro, in vivo, or even clinical data of PD effects. Again, these PD models can be linked to compartmental/ semi‐mechanistic PK or whole‐body PBPK models. In the latter, it is conceivable to specifically employ concentration‐time‐profiles of the organ of interest itself or of the subcompartment of the organ of interest to estimate the effect.^[^ ^216^, ^217^, ^218^ ^]^ While not in the scope of this review, it has to be mentioned that PK/PD modeling can also be employed for toxicity assessments early in development, frequently referred to physiologically based toxicokinetic (PBTK)/ toxicodynamic (TD) modeling. This enables to assess risks associated with exposure or high *C*_max_ levels. Similar to PBPK/PD modeling, PBTK/TD models can be constructed based on in vitro data or in vivo toxicology input.^[^ ^194^ ^,^ ^219^, ^220^, ^221^ ^]^

Like for compartmental PK models, also PD models can be either constructed as one (or more) compartments to study kinetics or as more complex interplays taking the physiological perspective of the pathogen, such as the pathway of infection or compartments inaccessible for treatment (frequently observed for intracellular pathogens like *S. aureus* ^[^ ^222^ ^,^ ^223^ ^]^) and the host's response into account. Frequently, still mainly compartmental PD models are used, whereas more recent quantitative systems pharmacology (QSP) or model‐based target pharmacology assessment (mTPA) evolve as concepts aiming to close the gap and add the physiological perspective also for PD (*vide infra*). Ultimately, this shall enable prediction of PD effect, not only in animal models but also for later clinical assessment, to increase probability of success for development and decrease attrition risks.^[^ ^177^ ^,^ ^206^ ^,^ ^224^, ^225^, ^226^, ^227^ ^]^ Certainly, these approaches will also improve compound selection for drug development as missing information will be added, otherwise not obtained from in vitro or in vivo experiments.

## 3 Examples for Adaptive Strategies for Development of Nontraditional Approaches

As outlined (*vide supra*), the development path for antivirulence agents^[^ ^161^ ^,^ ^228^, ^229^, ^230^, ^231^, ^232^ ^]^ is not as clear as for “traditional” antibacterials, as appropriate models need to be designed to provide a proof‐of‐concept.^[^ ^100^ ^]^ In the following, examples for antivirulence agents, that is, pathoblockers, as well as click‐to‐release systems as representatives of novel approaches will be given, outlining a potential adaptive strategy for their development.

Several QS inhibitors have been reported interfering with the Pqs system of *P. aeruginosa*, but only few report a detailed in vivo evaluation and provide a proof‐of‐concept.^[^ ^65^ ^,^ ^169^ ^,^ ^233^, ^234^, ^235^, ^236^, ^237^, ^238^ ^]^ It has been shown previously that QS inhibitors interfering with the Pqs system of *P. aeruginosa* can potentiate the efficacy of tobramycin to eradicate biofilms in vitro.^[^ ^236^ ^]^ Successful interference with the Pqs system is associated with a reduction of HHQ, HQNO, and PQS levels. Thus, these were employed as biomarkers to provide proof‐of‐target engagement during first in vivo studies.^[^ ^169^ ^,^ ^236^ ^]^ As no clear, established path is available on how to assess pathoblockers interfering with the Pqs QS system, Hamed and collegaues reported a study employing a modified and extended standard neutropenic thigh infection model with *P. aeruginosa* for efficacy testing of their PqsR inverse agonist. This modification of the standard “workhorse” model (*vide supra*) was driven by the hypothesis that a model extension together with a lower inoculum might cause upregulation of biomarkers, such as HHQ, HQNO, and PQS, and thus, enable to probe efficacy in vivo. Moreover, as antivirulence agents do not inherit antibacterial activity, a setup was chosen to potentiate the activity of standard‐of‐care, in that case tobramycin. That strategy was successful and a clear potentiation of a low‐dose, otherwise ineffective, tobramycin treatment was observed upon combination with the PqsR inverse agonist reported by Hamed and colleagues.^[^ ^32^ ^]^ Comparable studies in the field merely rely on in vitro assessment of biofilm disruption and/or in vivo evaluation with the established model organisms *Caenorhabditis elegans, Galleria mellonella*, or zebrafish. Albeit these systems enabling initial evaluation of activity in a more physiological context, they lack comparable mammalian physiology. Hence, these can just serve as a first step during discovery and development.^[^ ^169^ ^,^ ^239^, ^240^, ^241^ ^]^ Few other studies targeting QS evaluated their antivirulence agents in animal models.^[^ ^233^, ^234^, ^235^ ^,^ ^242^ ^,^ ^243^ ^]^ Kesarwani and colleagues assessed PqsBC inhibitors in a survival model using a thermal injury model in mice. They rather employed a preventive approach by pretreatment of the infection inoculum with the inhibitor prior to challenge so that, unfortunately, no information was obtained if an established infection was prevented.^[^ ^242^ ^]^ By contrast, Hentzer and colleagues provided a first validation for their compound targeting the LasR QS system using an acute lung infection model. Similar to previous studies, they also relied on assessment of QS signals, decreasing upon treatment with their antivirulence agent. Additionally, they were able to demonstrate that infection was cleared when treatment with pathoblocker started directly after challenge, hypothesizing that this was attributed to the host's immune response.^[^ ^233^ ^]^ After challenge, the infection needs to establish as reported previously.^[^ ^149^ ^,^ ^151^ ^,^ ^153^ ^]^ Thus, it can also be hypothesized that treatment with the pathoblocker reported by Hentzer et al. was successful because bacteria were hampered and unable to establish infection resulting in more efficient clearance by the immune system.^[^ ^233^ ^]^ By contrast, also Lesic and colleagues chose a therapeutic approach and employed a mouse burn model. They observed prolonged survival upon therapy, a reduction of bacterial dissemination into deeper tissue layers and a downregulation of biomarkers, representative of the mechanism of action of their QS inhibitor.^[^ ^234^ ^]^ Albeit not completely ameliorating infection outcome, Lesic and colleagues proved with distinct readouts that their pathoblockers represents a suitable strategy for improving therapeutic outcome and provided a proof‐of‐concept. Long and colleagues presented a dual inhibitor, which also targeted QS systems. Equally, they chose a wound model and observed potentiation of the effect of either low‐dose ciprofloxacin or low‐dose tobramycin in combination with their dual‐targeting antivirulence agent.^[^ ^235^ ^]^ Hence, they pursued a similar strategy as presented by Hamed and colleagues to also forecast the potential niche of pathoblockers as combination with standard‐of‐care.^[^ ^32^ ^,^ ^235^ ^]^ Antivirulence agents are thought to reduce evolvement of resistance. However, in in vitro experiments as well as in vivo experiments with *C. elegans*, it had been reported that resistance against Pqs inhibitors in adenosine‐rich environment had emerged.^[^ ^244^, ^245^, ^246^ ^]^ Thus, it was unclear if resistance occurs under in vivo conditions, harboring completely different nutrient supply and an associated distinct selective pressure.^[^ ^247^ ^,^ ^248^ ^]^ In this regard, the study of Starkey and colleagues is of importance. They chose to employ two distinct models for in vivo assessment of their Pqs regulon inhibitor. As expected because of the mechanism of action of the pathoblocker, no impact on bacterial burden was detected in both infection models. Moreover, they observed reduced mortality in the acute lung infection model upon pathoblocker treatment. Additionally, they assessed emergence of resistance, fortunately, not observed.^[^ ^243^ ^]^ Therefore, the study by Starkey et al. supported the hypothesis that antivirulence treatments, specifically Pqs QS interfering compounds, might be associated with less emergence of resistance. Like others,^[^ ^32^ ^,^ ^234^ ^,^ ^235^ ^]^ they also observed potentiation of the effect of standard‐of‐care when treating with the pathoblocker in the mouse burn model.^[^ ^243^ ^]^ So far, the impact of compounds targeting the Pqs QS system on a potential more efficient eradication of tolerant bacteria has not been systematically investigated yet.^[^ ^249^ ^]^ However, the study by Starkey et al. provides encouraging initial results with a positive impact on eradication of antibacterial‐tolerant pathogens.^[^ ^243^ ^]^ Finally, the few preclinical studies discussed herein provide a first guidance on how pathoblocker development can be designed. The majority of them has in common that a potentiation of effect of low‐dose antibacterial in combination with the pathoblocker was demonstrated. This forecasts that clinical development might consider superiority studies over standard‐of‐care as niche for antivirulence agents. When thinking about indications, the Pqs QS system is mainly involved in biofilms. Thus, biofilm‐associated infections might be the target indication for these approaches. The majority of the studies discussed herein in more detail, relied on rather acute models with immunocompetent animals, such as the wound, burn, or acute lung infection models.^[^ ^233^, ^234^, ^235^ ^,^ ^242^ ^,^ ^243^ ^]^ Only one study employed immunodeficient mice when using the neutropenic thigh infection model serving as a surrogate for efficiency in case of a hampered immune response.^[^ ^32^ ^]^ Typically, patients suffering from recurrent or difficult‐to‐eradicate *P. aeruginosa* infection might have at the same time a hampered immune response, such as in patients with bronchiectasis or cystic fibrosis (CF).^[^ ^250^ ^,^ ^251^ ^]^ Several animal models aiming to mimic the clinical situation of people with CF with *P. aeruginosa* infection have been developed.^[^ ^159^ ^,^ ^252^ ^,^ ^253^ ^]^ Consequently, to advance Pqs QS targeting pathoblockers toward clinical application, it will be necessary to prove their additive effects over standard‐of‐care alone in clinically relevant models, such as subacute or chronic lung or wound infection models, also reflecting the interplay with the host's immune response.

Similar to Pqs QS pathoblockers, antivirulence agents targeting LasB, a zinc‐dependent metalloprotease secreted by *P. aeruginosa*, represent an additional strategy for targeting the protein itself and not the regulator.^[^ ^170^ ^,^ ^254^ ^]^ Similar challenges exist as discussed for Pqs QS antivirulence agents. However, for LasB, IL‐1β is used as a surrogate biomarker. It is known that LasB cleaves pro‐IL‐1β to become activated IL1β. This has also been shown in in vivo lung infection models in mice and was proven with clinical isolates from ICU patients. Likewise, IL‐1β has been deployed as a biomarker in studies with distinct LasB inhibitors to provide indirect target engagement by reduced activated IL‐1β levels.^[^ ^255^, ^256^, ^257^, ^258^ ^]^ The majority of studies employed acute lung infection models to investigate the role of LasB. Beside reduction of IL‐1β levels upon LasB inhibitor treatment, bacterial load reduction was limited in total numbers, despite being significant.^[^ ^259^ ^]^ Konstantinović and colleagues explored a neutropenic lung infection model to probe their LasB inhibitor. Based on previous PK assessment and promising ELF concentrations, they directly administered the compound via nebulization into the lung. Similar to the Pqs QS system, they assayed impact of LasB inhibitor to potentiate low‐dose standard‐of‐care and observed a significant reduction of bacterial burden. At the same time, they determined LasB protein levels and observed less dissemination upon LasB inhibitor treatment providing a proof‐of‐target engagement, independent of IL‐1β.^[^ ^33^ ^]^ Considering the development path, LaFayette and colleagues suggested that acute infections might be more appropriate for positioning LasB inhibitors. They had observed that loss of the regulator LasR resulted in increased inflammation and subsequently lung damage suggesting that treatment of infections in people with CF might be associated with a higher risk of lung damage in the chronic setting.^[^ ^260^ ^]^ This complex regulation of LasB production in vitro and in vivo had been observed previously.^[^ ^170^ ^,^ ^261^ ^]^ Consequently, these examples emphasize that the indication of a pathoblocker has to be wisely chosen and closely aligned with the virulence mechanism in the targeted infection state.

A relatively recent approach are click‐to‐release systems. They have been probed in a neutropenic thigh infection model enabling dissemination for providing a proof‐of‐concept by demonstrating the release after click of the antibacterial colistin in the compartment affected by secondary seeding.^[^ ^44^ ^]^ In that study, the click‐to‐release system was administered locally via inhalation into the lung, as it had been observed previously that secondary seeding occurs,^[^ ^152^ ^]^ whereas the trigger for release was administered systemically. Although it is encouraging that the system works in a physiological environment,^[^ ^44^ ^]^ the development path is not clear yet. To provide a local release of the antibacterial, specific designs or infection indications need to be envisaged to avoid systemic release, which would render the click‐to‐release concept obsolete, as advantages would be diminished. Future studies will show how click‐to‐release systems position in the therapeutic landscape.

Finally, providing a proof‐of‐concept or a proof‐of‐target engagement for novel, unprecedented treatment approaches, as discussed in detail for the examples herein, remains a challenge. Animal models need to be adjusted or treatments need to be evaluated together with standard‐of‐care, adding additional complexity. However, in vivo models are essential to understand the complex interplay in the mammalian system with or without immune response, the pathogen, and the pathoblocker concept. Albeit not being a general rule, frequently standard models, such as urinary tract infection, neutropenic or acute lung infection, or skin infection models provide an initial good readout for estimation of PK/PD relationships when employed in a preventive setup. Examples include *inter alia S. aureus* α‐hemolysin‐targeting antibodies^[^ ^262^ ^,^ ^263^ ^]^ as well as a small molecule,^[^ ^35^ ^]^ a *S. aureus agr* QS inhibitor,^[^ ^264^ ^]^ antibodies targeting *P. aeruginosa* virulence factors,^[^ ^31^ ^,^ ^36^ ^]^ or inhibitors targeting uropathogenic *E. coli*.^[^ ^265^ ^]^ Employing this preventive setup for initial assessment helps to gather an understanding for the subsequent development path, requiring to also demonstrate therapeutic efficacy ultimately. This is critical to evaluate additive and positive effects of antivirulence interventions as well as to weigh risks and select an appropriate indication.^[^ ^266^ ^,^ ^267^ ^]^ Consequently, further investigation is needed to provide tailored models answering the questions posed by pathoblockers and facilitate their way toward clinical development.

## 4 New Developments in In Silico PK/PD Modeling for Evaluation and Elucidation of PK/PD Properties

The concept of (PB)PK/PD modeling has evolved over the recent decades as discussed herein (*vide supra*). Particularly in the field of antibacterial drug development, it is standard to employ modeling to describe and estimate the magnitude of PD effects or to elucidate the PK/PD index based on available data.^[^ ^86^ ^,^ ^87^ ^,^ ^206^ ^,^ ^268^, ^269^, ^270^, ^271^ ^]^ Several examples of successful modeling in the areas of (1) dose prediction of antibacterials to suppress regrowth and/or resistance, (2) clinical trial outcome forecasting using modeling, (3) PK/PD modeling in antiviral drug development, and (4) prediction and dose determination for PROTACs are provided in the following.

Compartmental PK/PD models have employed ELF instead of plasma for concentration‐time‐profiles in the indication of lung infections to determine potential dosing regimens to suppress resistance and/or regrowth of bacteria. Additionally, the probability of target attainment needed to achieve an effect in clinical application was calculated.^[^ ^83^ ^,^ ^85^ ^,^ ^207^ ^,^ ^208^ ^,^ ^272^, ^273^, ^274^ ^]^ Aranzana‐Climent, van Os, and colleagues went beyond typical PK/PD index or target attainment predictions and intended to forecast the outcome of a clinical trial retrospectively by using popPK modeling. They derived individual PK profiles and incorporated in vitro PD data to predict the probability of effect. Based on individual patient characteristics and a limited set of data from the enrollment of the study, they were able to identify factors associated with augmented risks.^[^ ^275^ ^]^ These results are encouraging as they might provide a step forward from the popPK perspective toward ‘virtual clinical trials’.^[^ ^276^ ^]^

Modeling and simulation had been introduced in the field of HIV research two decades ago. Albeit available simplified prediction of viral kinetics upon treatment, the field has benefitted from QSP models considering the complexity of biological processes including the dimension of occurrence of drug resistance and the combination of drugs available. By keeping this complexity and by intense modeling efforts, recommendations for improved therapeutic interventions with a focus on different populations, risk groups, drug resistance profiles, compliance, viremia level differences, or start of therapy were given.^[^ ^199^ ^,^ ^211^ ^,^ ^218^ ^,^ ^277^, ^278^, ^279^, ^280^, ^281^, ^282^ ^]^ Predictive models for the human system are essential, particularly in viral indications when in vivo models, mimicking the clinical situation, are scarce, or not available at all (*vide supra*). In the field of antibacterials, modeling is mainly used to foster the understanding of underlying effects and to provide an explanation. Therefore, models with reduced complexity are built. For antivirals, QSP approaches might be more suited to provide predictions for the cases outlined as biological processes and their connections are taken into account, whereas conventional PK/PD modeling considers a limited number of PD targets with a simplified setup.^[^ ^225^ ^,^ ^226^ ^]^ The example of Celgosivir in the indication for treatment of Dengue demonstrates that an in vivo proof‐of‐concept in animals is sometimes not sufficient,^[^ ^283^ ^]^ as it failed later in clinical trials because no effect on viremia was seen.^[^ ^284^, ^285^, ^286^ ^]^ Efforts are made to more closely capture the host's immune response as seen in humans in animal models as well. However, decrease of viremia levels due to the immune response is still not observed.^[^ ^287^ ^,^ ^288^ ^]^ Here, QSP models could help. A study showing efficacy of the natural product Soraphen A against Dengue in an in vivo model incorporated a PBPK/PD model reflecting viral kinetics in the different compartments as well as the differences in the animal and human immune response. This led to the conclusion that the compound would not be effective in humans, albeit showing efficacy in animals.^[^ ^177^ ^]^ However, a proper validation of the model presented in that study was lacking as no human PK and PD data were available so that the robustness of the model cannot be judged finally.

Compared to direct‐acting antivirals, PROTACs profit from PK/PD model development even more, if not being essential for these novel therapeutic approaches to avoid high attrition rates. Because of their event‐driven mechanism‐of‐action,^[^ ^69^ ^,^ ^70^ ^]^ the impact and the magnitude of the PD effect cannot be derived from in vitro assays requiring a modeling framework taking *inter alia* the extent of ternary complex formation as well as of degradation, the availability and expression of the E3‐ligase guiding proteasomal degradation as well as the half‐life of the protein‐of‐interest and related downstream effects into account.^[^ ^289^ ^]^ Apart from that, PROTACs come with additional PK challenges, not entirely reflected by current in vitro ADME assays (*vide supra*),^[^ ^112^ ^,^ ^290^, ^291^, ^292^ ^]^ essential to be incorporated into the PK/PD models which might require in vivo PK assessment to elucidate underlying mechanisms.^[^ ^289^ ^]^ Several PK/PD modeling frameworks have been established to date to allow to predict the extent of target protein degradation and to allow to forecast degradation as well as in vivo PD effects.^[^ ^293^, ^294^, ^295^, ^296^, ^297^ ^]^ As PROTACs differ substantially in their mechanism of action from conventional inhibitors, modeling can aid in prioritization of compounds based on binding affinities to select the best degrader within a series and avoid failure and is therefore strongly recommended to be implemented early in a program.^[^ ^289^ ^]^ To enable PK/PD models for PROTACs to provide guidance and a good strength of prediction, experimental data input from degradation, DMPK, ubiquitination, and binding assays as well as in case of viral infections, for viral dynamics, is required.^[^ ^71^ ^,^ ^289^ ^,^ ^298^, ^299^, ^300^ ^]^

All distinct PK/PD models have in common that they undergo iterative hypothesis‐generating, model refinement, and validation steps so that finally a robust model is obtained. With robust PK or PK/PD models describing underlying biological processes accurately, predictive performance of a drug for clinical testing can be provided with good confidence. In line with the concept of ‘virtual clinical trails’, ‘virtual populations’ are needed capturing characteristics of a comparable ‘real‐world’ target patient population.^[^ ^301^ ^]^ Although this concept has not been frequently tested, studies by Nakamura et al. as well as Sayama et al. in the field of oncology illustrate that compound as well as metabolite levels and consequences for therapy can be predicted with good confidence, enabling to improve dose recommendation for an optimized outcome.^[^ ^276^ ^,^ ^302^ ^]^

With the advent of artificial intelligence in other fields of biology and chemistry, this has also been started to be explored to improve PK/PD modeling. ADME predictions using molecular fingerprinting or PK/PD predictions using neural networks, for example, deep convolutional, to identify the PD target early in drug discovery have been investigated.^[^ ^303^, ^304^, ^305^, ^306^, ^307^ ^]^ These techniques bear the potential to accelerate and revolutionize drug development by reducing iterative cycles, including medicinal chemistry efforts. However, they rely on sufficiently large and diverse training data sets, also necessitating equally diverse validation sets.^[^ ^100^ ^]^ Recently, the concept of model‐based target pharmacology assessment (mTPA) was proposed taking advantage of the recent developments in using artificial intelligence applications. This concept intends to perform a retrospective analysis to identify the PK parameters needed to achieve the desired PD effect. It employs PBPK/PD modeling in conjunction with machine learning decision‐tree algorithms.^[^ ^224^ ^,^ ^308^ ^]^ This could help medicinal chemists early in discovery to guide efforts toward desired properties and specifies desirable PK properties for a specific PD effect, potentially contributing to lower attrition rates. However, to date, it has not been reported to be explored for the development of PROTACs, which could add an interesting perspective.

In summary, in particular in the field of antivirals as well as for novel treatment approaches, specifically for PROTACs, innovation and advancement of PK/PD modeling methods is observed, whereas in the field of antibacterials a wide range of knowledge as well as models describing processes are already available. Artificial intelligence methods have the perspective to reduce iterative cycles, advance QSP and mTPA approaches, and contribute to democratize the usage of PK/PD modeling during drug development further.

## 5 Summary and Outlook

Anti‐infective drug development harbors several challenges. For novel treatment approaches individualized solutions are demanded. PK/PD methods intend to provide tailored answers, depending on the drug class and the mechanism of action. Whereas this pathway is clearer and straightforward for direct‐acting antibacterials and antivirals, new paths are being explored for pathoblockers, click‐to‐release‐systems, and PROTACs. For pathoblockers, that is, antivirulence agents, PK considerations follow typical small molecule development, but PK/PD relationships need to be elucidated depending on the mechanism of action, frequently requiring appropriate biomarkers. By contrast, click‐to‐release systems require orchestrating PK of the respective players to achieve the PD effect. PROTACs pose questions on PK and PD, which need to be solved in conjunction with PK/PD modeling. As described herein, the field of PK/PD offers an arsenal of methods, constantly evolving, for providing a mechanistic understanding for translation from bench to beside, strongly encouraging broader implementation of PK/PD modeling.

In summary, in vitro, in vivo, and in silico (PK/PD modeling) methods are different parts to form the PK/PD mosaic, indispensable for modern drug development. Future method developments, specifically in the field of PK/PD modeling, are expected. Moreover, PK/PD relationships will need to be elucidated for novel approaches in anti‐infective development. This will contribute to define selection criteria and shape go‐/no‐go‐decisions, to ultimately reduce attrition and accelerate development.

## Conflict of Interest

The author declares no conflict of interest.
